# Achieving High Strength in Mg-0.7Sm-0.3Zr Alloy via Room-Temperature Rotary Swaging: Radial Gradient Microstructure and Grain Refinement Mechanisms

**DOI:** 10.3390/ma18133199

**Published:** 2025-07-07

**Authors:** Jie Liu, Yuanxiao Dai, Zhongshan Li, Yaobo Hu

**Affiliations:** 1 College of Undergraduate Education, Shenzhen Polytechnic University, Shenzhen 518055, China; 2 College of Materials Science and Engineering, Chongqing University, Chongqing 400044, China; dyx@stu.cqu.edu.cn (Y.D.); lizhongshan076@163.com (Z.L.); yaobohu@cqu.edu.cn (Y.H.)

**Keywords:** magnesium alloy, rotary swaging, grain refinement, microstructure

## Abstract

Room-temperature rotary swaging was conducted on microalloyed high-ductility Mg-0.7Sm-0.3Zr alloy rods to investigate microstructural and mechanical variations across different swaging passes and radial positions. The results indicate that following room-temperature rotary swaging, the alloy rods exhibit a large number of tensile twins and low-angle grain boundaries, leading to significant grain refinement. After two swaging passes, the microstructure exhibits a pronounced radial gradient, characterized by progressively finer grain sizes from the core to the edge regions, with a hardness difference of 3.8 HV between the edge and the core. After five swaging passes, the grain size was refined from an initial 4.37 μm to 2.02 μm. The yield strength and ultimate tensile strength of the alloy increased from 157 MPa and 210 MPa in the extruded state to 292 MPa and 302 MPa, respectively. This observed strengthening is primarily attributed to grain refinement, dislocation hardening, and texture strengthening, with grain refinement playing the dominant role. The grain refinement process during rotary swaging can be divided into two stages: in the initial stage, coarse grains are subdivided by tensile twinning; in the later stage, high-stress-induced grain boundary bulging leads to new dynamic recrystallization, further refining the microstructure.

## 1. Introduction

Magnesium alloys, recognized as the lightest metallic structural materials, offer significant advantages such as low density and high specific strength, which present extensive application opportunities in aerospace, automotive lightweighting, biomedical devices, and consumer electronics [1,2,3]. Furthermore, their excellent recyclability aligns well with modern industrial demands for sustainable development [4]. However, the hexagonal close-packed crystal structure of magnesium inherently limits its deformability at room temperature, significantly constraining its broader applications [5,6]. Additionally, magnesium alloys exhibit lower intrinsic strength compared to other structural metals, such as aluminum and copper [7]. Consequently, achieving a simultaneous enhancement of both strength and ductility through process optimization remains one of the most critical challenges in current magnesium alloy research.

The primary methods for strengthening magnesium alloys include solid solution strengthening, precipitation strengthening, grain refinement strengthening, and work hardening [8,9,10,11]. The addition of rare earth (RE) elements can simultaneously enhance both strength and ductility, with mechanisms involving both solid solution strengthening and second-phase precipitation strengthening [12]. However, while a high RE content promotes beneficial second-phase precipitation, it also significantly increases material costs [13]. In contrast, grain refinement strengthening (Hall–Petch effect) has been shown to be the most effective method for improving the strength-toughness synergy in magnesium alloys [14,15].

Severe plastic deformation (SPD) techniques are among the most critical methods for achieving grain refinement [16,17]. Among these techniques, rotary swaging—an efficient and continuous SPD process—induces intense shear deformation through multi-pass radial forging, resulting in remarkable grain refinement and enhancement of mechanical properties [18,19]. Compared to other SPD processes, such as equal-channel angular pressing [16] and high-pressure torsion [20], rotary swaging offers superior processing efficiency and continuous forming capability, making it particularly suitable for the industrial-scale production of long-length rods and tubes [21].

Rotary swaging can be classified into three categories based on processing temperature variations: room-temperature, warm, and cryogenic swaging [22,23,24]. Room-temperature rotary swaging is applicable to certain magnesium alloys that possess adequate ductility; however, it tends to induce strong basal fiber textures during deformation, resulting in anisotropy in mechanical properties [17,18]. Wang et al. [22] demonstrated significant effects of room-temperature rotary swaging on both grain size and secondary phases in the Mg-Al-Mn-Zn-Ca alloys, reporting improvements of 74 MPa in ultimate tensile strength (UTS) and 161 MPa in yield strength (YS) after 13 passes. Warm rotary swaging (typically 150–300 °C) activates non-basal slip systems and promotes dynamic recrystallization (DRX), thereby enhancing the capability for homogeneous deformation [23,25]. Comparative studies by Li et al. [25] on the AZ80 magnesium alloy revealed that room-temperature rotary swaging more frequently caused premature failure, whereas specimens subjected to warm rotary swaging developed radial gradient structures from edge to core, exhibiting superior strength-ductility synergy. Cryogenic rotary swaging (below liquid nitrogen temperature) further suppresses dynamic recovery (DRV) while increasing dislocation density, achieving more pronounced grain refinement and strengthening [21]. Chen et al. [21] observed abundant nanoscale subgrains in the AZ31 alloy cooled with liquid nitrogen, with cryogenic processing demonstrating superior grain refinement efficiency and greater strength enhancement compared to room-temperature rotary swaging.

Despite demonstrating remarkable strengthening effects, rotary swaging still presents several unresolved issues. First, the relationship between processing parameters (deformation degree, pass interval, temperature) and DRX behavior/grain refinement extent remains unclear. For instance, the high-strength AZ80 alloy tends to experience premature failure during low-temperature swaging, while the medium-strength AZ31 alloy exhibits better adaptability to low-temperature processing [21,25]. However, the nanocrystalline grains formed in the AZ31 alloy during cryogenic processing deviate from the expected DRX kinetics [21,23,25]. Furthermore, the strengthening mechanism of rotary swaging remains controversial. Most studies emphasize that the nanograins formed after swaging are the primary contributors to alloy strengthening [21]. However, significant heterogeneity persists in the post-swaged microstructure. In practice, work hardening likely serves as the dominant strengthening mechanism in swaging, as the mechanical curves of most swaged specimens exhibit no discernible work-hardening stage [25]. Consequently, systematic investigation is imperative to elucidate how swaging parameters govern microstructural evolution and mechanical properties in magnesium alloys.

This study focused on the microalloyed high-ductility Mg-0.7Sm-0.3Zr alloy to systematically investigate the microstructural evolution and mechanical property development during multi-pass room-temperature rotary swaging. Through an integrated analysis of Electron Backscatter Diffraction (EBSD), metallographic characterization, and mechanical testing, the intrinsic correlation between swaging-induced deformation mechanisms and performance optimization was elucidated. After rotary swaging, the Mg-0.7Sm-0.3Zr alloy exhibits a remarkable improvement in strength, adequate for most structural applications. Furthermore, the analysis of varying passes of swaging passes contributes to the development of the theoretical framework for rotary swaging of magnesium alloys.

## 2. Materials and Methods

The Mg-0.7Sm-0.3Zr (wt.%) alloy used in this study was prepared via semi-continuous casting, producing an ingot with a diameter of 80 mm. The Sm element is relatively cost-effective, while Zr acts as an efficient grain refiner in magnesium alloys [26]. The ingot was homogenized at 410 °C for 40 min in a resistance heating furnace, followed by direct extrusion using an XJ-500 horizontal extruder. The extrusion was conducted at 410 °C with a speed of 5 m/min and an extrusion ratio of 16.8:1. The resulting 20 mm-diameter extruded rods were immediately water-quenched to room temperature.

**Figure 1:**
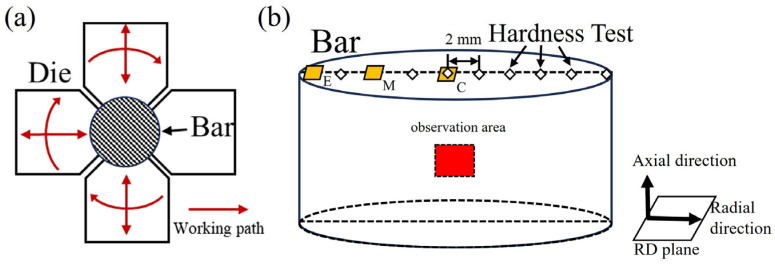
(**a**) Schematic illustration of rotary swaging; (**b**) hardness testing locations (diamond symbols) and EBSD observation areas—yellow-coded plane indicates the transverse section, red-coded plane denotes the longitudinal section.

The extruded rods were machined to remove surface irregularities, yielding a rod with a diameter of 19.2 mm for subsequent room-temperature rotary swaging. The swaging process employed four dies rotating at high speed around the rod while applying short-stroke rapid impacts radially. A schematic of the swaging die configuration is presented in Figure 1a. The true strain after each swaging pass was calculated using the following equation:

(1) $$\varepsilon =\ln(S_{0}/S_{i})$$

where $S_{0}$ represents the initial rod diameter and $S_{i}$ denotes the rod diameter after i passes. In this study, the extruded rods underwent five consecutive swaging passes, with diameter reductions of 0.4 mm per pass for the first two passes and 1.2 mm per pass for the subsequent three passes. The corresponding calculated true strain values are presented in Table 1. The specimens were designated according to their number of swaging passes.

EBSD characterization was conducted using a JEOL JSM-7800F field-emission scanning electron microscope (Japan Electron Optics Laboratory, Tokyo, Japan) equipped with a NordlysMax2 EBSD detector (Oxford Instruments, Oxford, UK). Cylindrical specimens with a height of 4 mm were prepared via wire electrical discharge machining and bisected along the base diameter to enable microstructural characterization of the cross-sectional plane (indicated by the red plane in Figure 1. To comparatively analyze the microstructural heterogeneity from the surface to the core of the bar, additional characterizations were conducted on the radial plane at the central, edge, and mid-radius (1/2 R) positions, as demarcated by the yellow regions in Figure 1b.

Specimen surfaces were mechanically ground using SiC abrasive papers (400–2000 grit) to eliminate macroscopic scratches, followed by electropolishing in commercial AC2 solution under 20 V for 150 s. EBSD data acquisition parameters included 20 kV accelerating voltage, 15 μA probe current, and 0.5 μm step size. The acquired datasets were processed using the MTEX toolbox (version 5.10.2) based on MATLAB (version R2024b) and the AZtecCrystal software (version 2.1). Grain size was calculated using EBSD data, with over 1000 grains analyzed per condition. The equivalent circular diameter was determined from pixel data to represent grain size, ensuring the reliability of the grain size statistics. Metallographic specimens were chemically etched in 4% nitric acid–alcohol solution for 15–20 s after electropolishing, rinsing with anhydrous ethanol before optical microscopy.

The rotary-swaged samples were cut into thin discs with a diameter of 3 mm and a thickness of 0.1 mm. These discs were first ground with sandpaper to a thickness below 50 μm and then thinned using a Gatan 695 ion milling system. The specific thinning parameters were as follows: (i) high-voltage milling at 5 kV with an incident angle of 6° until perforation and (ii) subsequent fine polishing at 3 kV with a reduced angle of 3° for 30 min. TEM observations were primarily conducted using an FEI Titan G2 60–300 aberration-corrected transmission electron microscope.

Vickers hardness measurements were performed using an HV-1000TM/LCD hardness tester (Jinan Kason Testing Equipment Co., Ltd., Jinan, China). The radial surfaces of the cylindrical samples were also mechanically ground to ensure that they were flat and smooth. Testing parameters comprised a 500 gf applied load with a 10 s dwell time. Hardness checkpoints were aligned radially at 2 mm intervals across the specimen surface, as schematically depicted in Figure 1b. The hardness value was tested 3 times per checkpoint and averaged.

Tensile mechanical properties were evaluated using a SANSI CMT-5105 universal testing machine (Sansi Eternal Technology Co., Ltd., Wenzhou, China). Specimen dimensions were designed by Chinese National Standard GB/T 228.1-2021 [27], featuring a dog-bone shape with a 25 mm gauge length and 5 mm diameter. All tests were conducted at a constant strain rate of 2.0 × 10^−3^ s^−1^, with loading direction aligned parallel to the initial extrusion direction (ED) and the axial direction (AD) of the rotary swaging bar (Figure 1). To minimize test errors, at least three parallel samples were used for each set of passes.

## 3. Results

### 3.1. Microstructure of the Extruded Mg-Sm-Zr Alloy

**Figure 2:**
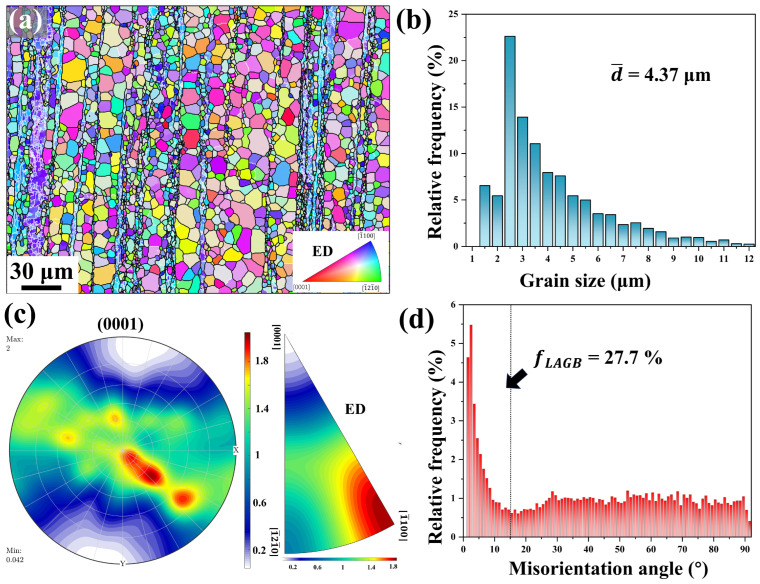
Initial microstructure of the extruded Mg-0.7Sm-0.3Zr alloy: (**a**) IPF map; (**b**) grain size distribution histogram; (**c**) (0001) PF and IPF with the extrusion direction as the reference axis; (**d**) misorientation angle distribution histogram.

The extruded Mg-0.7Sm-0.3Zr alloy exhibited a distinct heterogeneous microstructure, including equiaxed recrystallized grains, elongated non-recrystallized grains, and fine-grained bands aligned along the ED, as shown in Figure 2a. This unique structure arises from Zr’s suppression of DRX, as previously demonstrated [28]. The alloy displayed an average grain size ($\bar{d}$) of 4.37 μm, where smaller initial grains enhance refinement during deformation by increasing grain boundary density, promoting finer recrystallized grains [29].

Sm addition induced RE texture characteristics, with the (0001) pole figure (PF) showing a maximum intensity of 2.0 multiples of uniform density (mud). However, due to the high fraction of non-recrystallized grains, the Inverse pole figure (IPF) revealed predominant [1$\bar{1}$00] orientation alignment. Residual dislocations and subgrain boundaries within non-recrystallized regions contributed to a high proportion of low-angle grain boundaries (LAGBs, $f_{LAGB}=$ 27.7%). Meanwhile, high-angle grain boundaries (HAGBs) were uniformly distributed, consistent with Figure 2d.

### 3.2. Mechanical Properties

**Figure 3:**
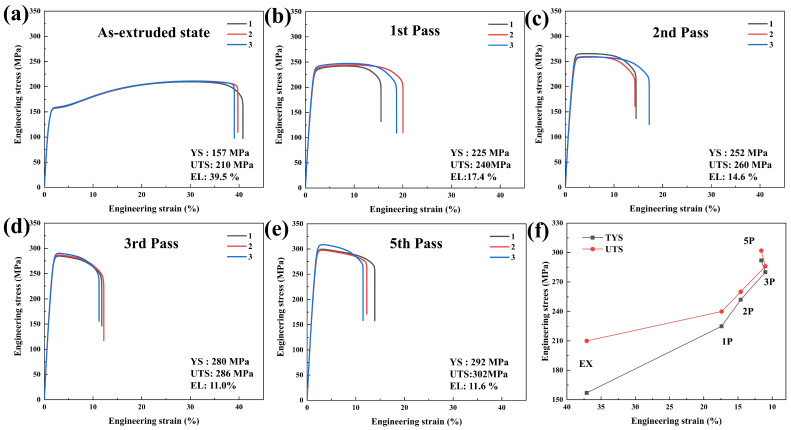
Engineering stress–strain curves of the Mg-0.7Sm-0.3Zr alloy, Samples 1, 2, and 3 in the figure are three parallel specimens under the same condition.: (**a**) as-extruded state; (**b**) first pass; (**c**) second pass; (**d**) third pass; (**e**) fifth pass; and (**f**) evolution of the average mechanical properties of the three parallel specimens during the rotary swaging process.

The tensile engineering stress–strain curves of rotary-swaged Mg-0.7Sm-0.3Zr alloy through different processing passes are presented in Figure 3, with average mechanical properties of triplicate samples for each pass displayed in the bottom-right inset. The as-extruded alloy exhibited notable plastic deformation capacity, showing an average elongation (EL) at break of 39.5% with tensile yield strength (TYS) and ultimate tensile strength (UTS) of 157 MPa and 210 MPa, respectively. Significant work hardening was observed in the original extruded specimen, whereas engineering stress–strain curves after rotary swaging displayed rapid TYS enhancement without distinct work hardening stages.

Rotary swaging increased TYS and UTS to 225 MPa and 240 MPa after the first pass (68 MPa and 30 MPa improvements over the extruded state). Subsequent passes further enhanced strengths to 252 MPa (TYS) and 260 MPa (UTS) with reduced elongation (14.6%) after the second pass. The third pass yielded reduced hardening efficacy (TYS = 280 MPa, UTS = 286 MPa) and elongation (11.0%). Notably, fifth-pass specimens demonstrated unexpected elongation recovery (11.6%, Figure 3f) despite accumulated residual dislocations causing continuous ductility decline. This anomaly suggests partial ductility restoration through DRX during progressive swaging.

The Mg-1Zn-0.2Ca (wt.%) alloy [30] processed by multiple passes of ECAP developed a bimodal grain structure, with fine grains averaging 5.0 μm. The alloy exhibited a UTS of 283 MPa and an elongation of 25%. Similarly, a Mg-1.8Zn-0.7Mn-0.02Fe-0.02Ni alloy [31] processed by four passes of ECAP achieved a UTS of 227 MPa and an improved elongation of 27%, with an average grain size of 5.4 μm. Chen et al. [24] investigated the microstructural evolution and mechanical property changes in pure Mg and Mg-Gd alloys after five passes of cold rotary swaging (true strain of 24%). The results showed that rotary swaging led to a decrease in strength but an improvement in ductility for pure magnesium. In contrast, for the Mg-1Gd alloy, the UTS increased significantly from 180 MPa to 329 MPa, while the elongation to fracture decreased from 32.6% to 13.4%. These results demonstrate the significant strengthening effect of SPD on microalloyed magnesium alloys. In our study, after five passes of rotary swaging, the Mg-0.7Sm-0.3Zr alloy exhibited a UTS and TYS of 292 MPa and 302 MPa, respectively. The swaged samples contained a high density of dislocations, indicating a strong dislocation strengthening effect, which will be further discussed in the subsequent sections.

**Figure 4:**
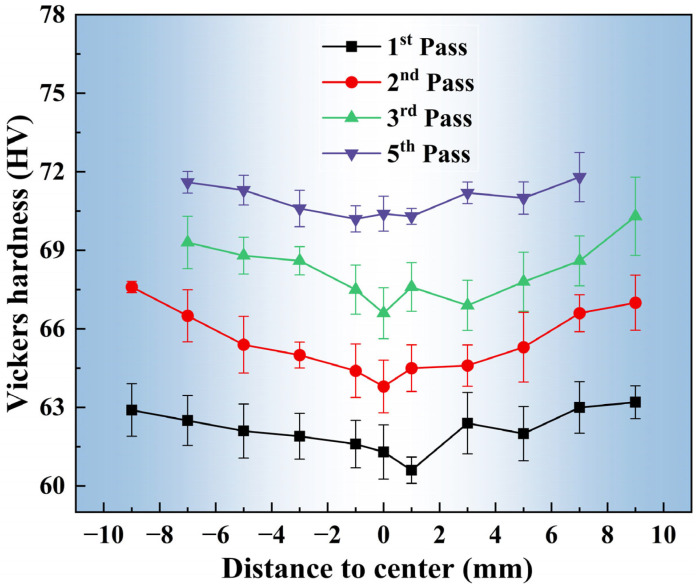
Radial Vickers hardness measurements (2 mm intervals) of as-extruded Mg-0.7Sm-0.3Zr alloy after multi-pass rotary swaging.

Figure 4 displays the radial hardness distribution across the cross-sections of Mg-0.7Sm-0.3Zr alloy bars processed by different rotary swaging passes. Post-swaging hardness differentiation between the core and edge regions is evident, with lower hardness in the core and enhanced edge hardening. After the first pass, edge hardness reached 63 HV versus 61 HV at the core. The greatest disparity (3.8 HV) occurred after the second pass. This core-edge hardness disparity diminished with increasing swaging passes, achieving uniform hardness distribution (average 71.6 HV) after the fifth pass.

This radial hardness distribution contrasts sharply with Chen et al.’s [21] AZ31 swaging results, where core hardness consistently exceeded edge values, with widening gaps across passes. These discrepancies may correlate with the specific pass reduction ratios selected during swaging and the single-strike impact force configuration of the rotary forging apparatus. However, mechanical analysis [32] confirms that edge regions undergo greater deformation-induced strengthening, aligning with our observed Mg-0.7Sm-0.3Zr alloy hardness progression. The second-pass specimen (maximized core-edge differentiation) was selected for subsequent cross-sectional microstructure characterization.

### 3.3. Microstructure Evolution During Swaging

**Figure 5:**
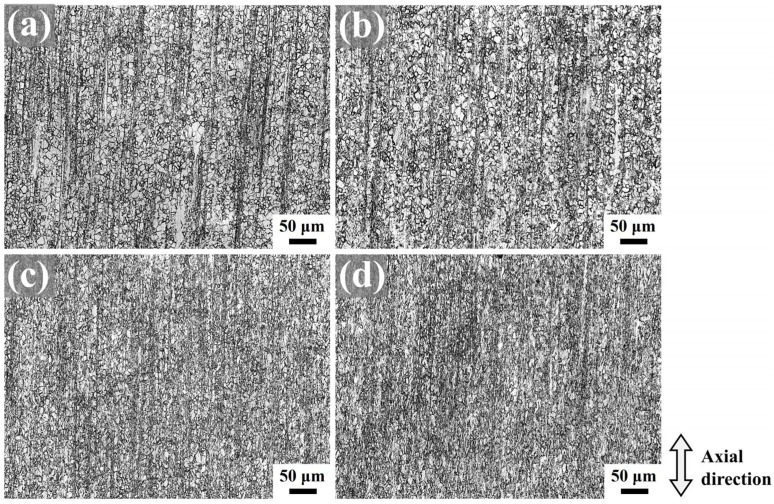
Metallographic micrographs of as-extruded Mg-0.7Sm-0.3Zr alloy after multi-pass rotary swaging: (**a**) first pass; (**b**) second pass; (**c**) third pass; and (**d**) fifth pass.

Figure 5 presents the optical microstructures of as-extruded Mg-0.7Sm-0.3Zr alloy subjected to rotary swaging with varying passes, observed at the surface location outlined by the red frame in Figure 1b. After the first pass, the alloy retained its equiaxed grain structure with alternating non-recrystallized grains and fine-grained bands, while tensile twins were identified within certain equiaxed grains. Incremental grain refinement occurred with increasing swaging passes, accompanied by numerous fragmented fine grains aligned along the axial direction.

By the third pass, the fine-grained bands and non-recrystallized grains gradually diminished, though retained some large equiaxed and elongated grains resulting from axial grain stretching under radial compressive stresses during swaging. Darker regions observed in fifth-pass micrographs correspond to areas with higher grain/subgrain boundary densities, confirming effective grain refinement. Notably, the microstructure exhibited pronounced heterogeneity post-swaging deformation, with residual coarse grain structures persisting within the processed material.

**Figure 6:**
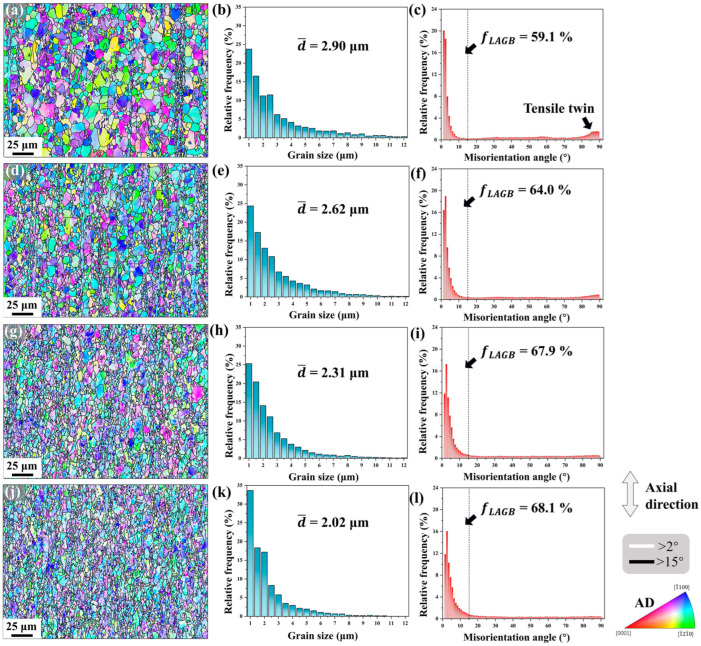
IPF maps, grain size distributions and misorientation angle distributions of Mg-0.7Sm-0.3Zr alloy during multi-pass rotary swaging: (**a**–**c**) first pass; (**d**–**f**) second pass; (**g**–**i**) third pass; and (**j**–**l**) fifth pass.

EBSD analysis was conducted on the microstructure along the processing direction of Mg-0.7Sm-0.3Zr alloy, as shown in Figure 6. The analyzed region measured 240 × 180 μm, with indexing rates exceeding 80% for all swaged samples, ensuring comprehensive and accurate microstructure characterization. In the IPF maps, LAGBs (2–15°) are marked with white lines, while HAGBs (>15°) are denoted by black lines, referenced to the axial direction. With increasing swaging passes, enhanced blue/green grain coloration in IPF maps indicates a growing proportion of grains with c-axes perpendicular to the axial direction.

After the first rotary swaging pass, abundant tensile twins formed in the Mg-0.7Sm-0.3Zr alloy while grain morphology remained largely unchanged. In Figure 6a, a large number of fine grains aligned axially in a streamline pattern are observed in the right region, corresponding to the fine-grained band structure formed after extrusion. This linear distribution of the fine-grained band remains after one swaging pass. Meanwhile, tensile twins are not activated within the unrecrystallized grains in Figure 6a, indicating that unrecrystallized grains with fiber-texture orientations are less prone to tensile twinning during swaging, consistent with observations by Wang et al. [22].

After the first swaging pass, the average grain size of the alloy decreased from 4.37 μm to 2.90 μm, as shown in Figure 6b, primarily due to the activation of tensile twins and their grain-segmentation effect. With increasing swaging passes, the average grain size of the swaged samples progressively reduced to 2.02 μm. In subsequent swaging passes, activation of tensile twins is scarcely observed. Therefore, tensile twinning exerts minimal influence on the samples during these stages, while grain refinement likely originates from alternative mechanisms. A striking observation is that the frequency of grains around 1 μm remained approximately 25% in specimens subjected to 1, 2, and 3 swaging passes, but increased to 33.7% in the fifth pass sample. This suggests that the fifth pass swaging likely promoted DRX behavior, generating numerous new fine dynamically recrystallized grains in the alloy.

Figure 6 also presents the misorientation angle distributions of the alloy after different swaging passes. The changes in these distributions provide insight into the underlying reasons for the multi-pass swaging-induced grain refinement. Following the first swaging pass, the LAGB proportion abruptly increased to 59.1%, accompanied by a peak near 86.3° corresponding to tensile twins. As swaging progressed, the LAGB proportion continued rising with diminishing growth rates, while the twin-related peak weakened and eventually disappeared. LAGBs fundamentally consist of dislocation arrays, geometrically manifesting ordered dislocation arrangements [33]. With increasing swaging passes, the LAGB proportion in the alloy rises gradually. Notably, the LAGB proportion in the fifth pass swaged sample shows minimal disparity compared to the third pass counterpart, suggesting that dislocation storage within the grains may have approached saturation. Additionally, the finer grain size observed in the fifth pass swaged specimen implies that deformation energy from swaging promotes dislocation slip/climb in pre-existing LAGBs. This process introduces additional dislocations into grain boundaries, increases misorientation angles, and ultimately facilitates the formation of HAGBs.

**Figure 7:**
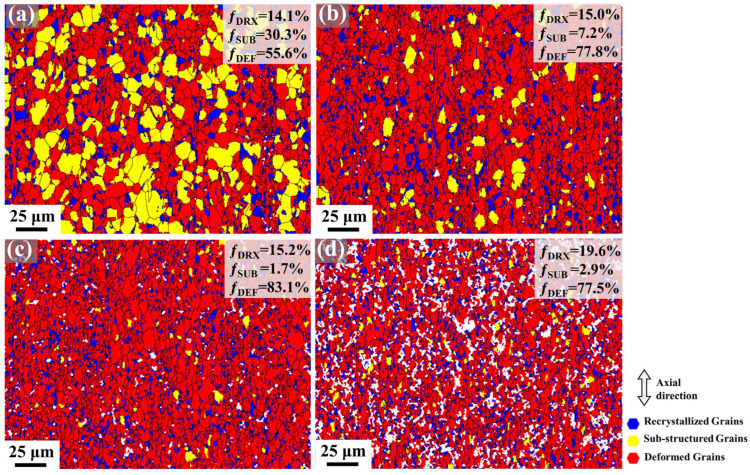
Def-Rex maps in Mg-0.7Sm-0.3Zr alloy bars during multi-pass rotary swaging: (**a**) first pass; (**b**) second pass; (**c**) third pass; (**d**) fifth pass.

Furthermore, recrystallization behavior in the samples elucidates the transition between LAGBs and HAGBs. Figure 7 presents the deformation-recrystallization (Def-Rex) grain distribution maps of the Mg-0.7Sm-0.3Zr alloy after different swaging passes. Grain orientation spread (GOS) was employed to characterize plastic deformation levels or recrystallization states: blue (GOS < 2°), yellow (2° < GOS < 5°), and red (GOS > 5°) represent recrystallized grains, substructured grains, and deformed grains, respectively. Notably, recrystallized grains in 1–3 pass samples likely include some non-deformed grains, though no further distinction was made in this study.

In the first pass swaged sample, a substantial number of substructured grains are observed, with the recrystallized grain fraction at 14.1%. These grains, characterized by smaller sizes, likely remain undeformed plastically. This indicates that macroscopic deformation preferentially initiates in larger grains at the microscopic level. As swaging passes increase, the fraction of deformed grains progressively rises, while substructured grains correspondingly diminish. Notably, the DRX grain fraction remains largely unchanged. Figure 6 demonstrates a sharp increase in the frequency of grains around 1 μm after 5 swaging passes. Concurrently, Figure 7 reveals no growth in the LAGB proportion. These findings collectively suggest pronounced DRX in the fifth pass specimen. Crucially, Figure 7d corroborates this: the DRX grain fraction increases from 15.2% (third pass) to 19.2% (fifth pass) with reduced deformed grains. This implies that accumulated dislocation storage lowers the critical temperature for DRX nucleation. Under high-stress conditions during multi-pass swaging, extensive DRX nucleation occurs, simultaneously refining grain size and consuming partial dislocations.

**Figure 8:**
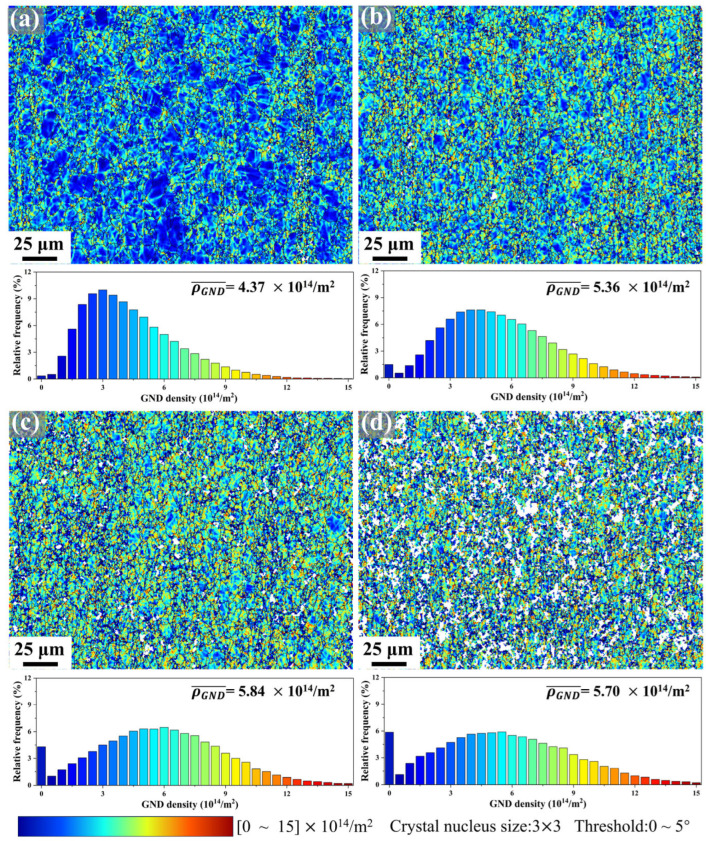
GNDs mapping and distribution in Mg-0.7Sm-0.3Zr alloy bars during multi-pass rotary swaging: (**a**) first pass; (**b**) second pass; (**c**) third pass; (**d**) and fifth pass.

Figure 8 presents geometrically necessary dislocation (GND) distribution maps and corresponding density analyses for Mg-0.7Sm-0.3Zr alloy bars subjected to incremental rotary swaging passes. The GND density ($\rho_{GND}$) was calculated directly using AZtecCrystal software with a Crystal nucleus size of 3 × 3 pixel, misorientation angle cutoff of 0–5°, and density range of 0–15 × 10^14^/m^2^.

Notably, the dominant color in the GND maps progressively transitions from blue to yellow-green with increasing swaging passes, signaling systematic dislocation density escalation. Frequency distributions and mean GND densities ($\bar{\rho_{GND}}$) reveal consistent growth from 4.37 × 10^14^/m^2^ (first pass) to 5.84 × 10^14^/m^2^ (third pass). However, the fifth pass specimen exhibits a slight reduction to 5.70 × 10^14^/m^2^, aligning with Figure 7 observations. This reversal reflects DRX activation triggered by intense dislocation accumulation during later stages. The newly formed fine DRX grains refine the microstructure while consuming partial dislocations, leading to reduced dislocation density in the fifth pass swaged specimen. However, this contributes to enhanced grain refinement strengthening effects. Concurrently, dislocation consumption during recrystallization induces partial softening, while fresh dislocations are stored within new DRX grains, restoring plastic deformation capacity. This dual mechanism explains the improved elongation in fifth pass specimens (Figure 3), illustrating strength-ductility synergy achieved through process optimization.

**Figure 9:**
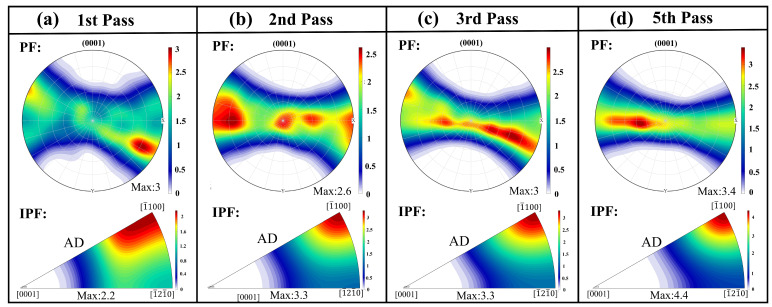
PFs and IPFs of Mg-0.7Sm-0.3Zr alloy bars after multi-pass rotary swaging.

Figure 9 illustrates the texture evolution of the Mg-0.7Sm-0.3Zr alloy bar during swaging. The upper and lower panels present the (0001) PFs and IPFs after different swaging passes, respectively. In the PFs, the X-axis corresponds to the radial direction of the bar, while the Y-axis represents its axial direction. The as-extruded alloy exhibits a RE texture, demonstrating a maximum texture intensity of merely 2.0 mud in the PF (Figure 2). After the first swaging pass, the (0001) PF pole density concentrates toward the horizontal direction (radial axis), with enhanced clustering upon subsequent passes. The IPFs simultaneously reveal a gradual alignment of the [1$\bar{1}$00] direction with the bar’s axial direction. Consequently, progressive texture evolution from a RE orientation to a (0001) [1$\bar{1}$00] fiber texture occurs with increasing swaging passes.

Activation and propagation of tensile twins during the first and second passes (Figure 6) predominantly drive this texture modification. Significant texture transformation observed after the first pass aligns with Zheng et al. [28], where tensile twinning reorients non-basal grains into basal orientations, forming basal fiber textures. Subsequent texture intensification likely stems from grain rotation mediated by widespread basal/non-basal slip activation, enabling small-angle lattice realignment along Taylor axes [34,35]. With increasing swaging passes, the basal texture intensity of the alloy progressively strengthens, accompanied by enhanced orientation concentration. During axial tensile testing, this texture configuration impedes basal slip activation. Consequently, the pronounced basal texture induces a significant texture strengthening effect, elevating the alloy’s strength while reducing ductility.

### 3.4. Microstructural Evolution Along the Radial Direction

**Figure 10:**
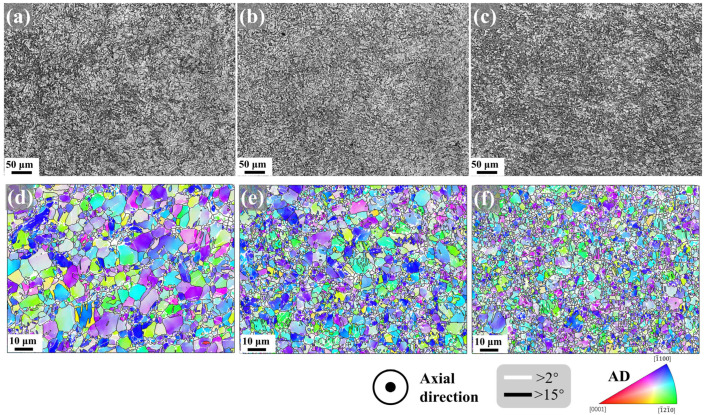
Optical microscopy and IPF maps at different radial positions of Mg-0.7Sm-0.3Zr alloy after two passes rotary swaging: (**a**,**d**) center; (**b**,**e**) mid-radius; (**c**,**f**) edge.

During swaging, the deformation degree of the material gradually decreases from the radial edge to the center. As shown in Figure 4, the central region exhibits lower hardness values, whereas the edge region displays higher hardness. Accordingly, the microstructure across the radial direction of the cross-section in the second pass swaged Mg-0.7Sm-0.3Zr alloy was characterized. Figure 10 presents the optical micrographs and IPF maps of the central region, mid-radius, and edge along the cross-sectional radial direction.

As shown in Figure 10d, no evident deformation traces are observed in the central region, where most grains retain an equiaxed morphology. Activation of tensile twins is notably visible only within larger equiaxed grains. In the mid-radius zone, a heterogeneous structure emerges, characterized by intermixed fragmented fine grains and coarser equiaxed grains, with tensile twin activation also detected in larger grains. Near the edge, coarse equiaxed grains undergo further fragmentation, yielding a greater population of refined grains, while similar activation of tensile twins is identified. Collectively, the Mg-0.7Sm-0.3Zr alloy exhibits a gradient microstructure along the radial direction from edge to center: the edge experiences more pronounced deformation with significant grain refinement, whereas the less-deformed central region retains numerous coarse grains.

**Figure 11:**
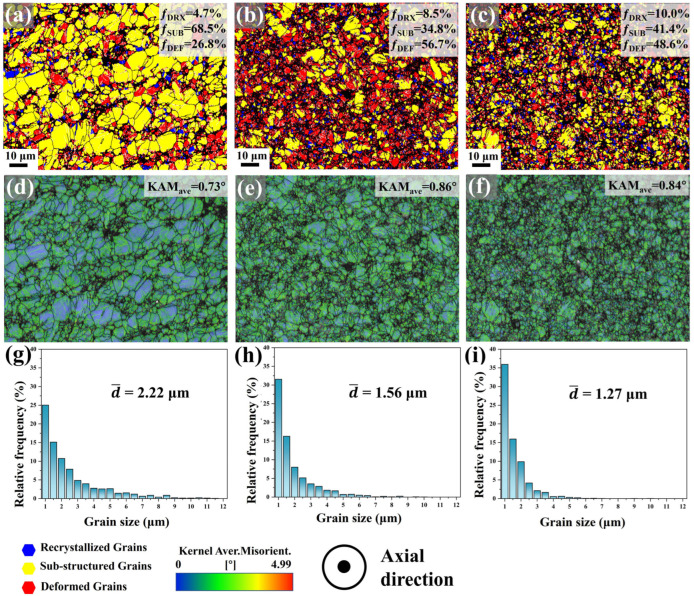
Def-Rex maps, kernel average misorientation (KAM) maps and grain size distribution at different radial positions of Mg-0.7Sm-0.3Zr alloy after two passes rotary swaging: (**a**,**d**,**g**) center; (**b**,**e**,**h**) mid-radius; (**c**,**f**,**i**) edge.

Figure 11 presents the Def-Rex maps, kernel average misorientation (KAM) maps, and grain size distribution profiles across the radial direction of the second pass swaged specimen. In the central region, the microstructure predominantly consists of substructured grains (68.5%), with lower fractions of recrystallized grains (4.7%) and deformed grains (26.8%). This area exhibits a relatively low average KAM value (0.78°) and an average grain size of 2.22 μm. Significant microstructural evolution occurs at mid-radius: the deformed grain fraction sharply increases to 56.7%, accompanied by a moderate rise in recrystallized grains (8.5%) and a reduction in substructured grains (34.8%). Near the edge, further recrystallization progress is evident, with substructured grains decreasing to 41.4%, dynamically recrystallized grains increasing to 10.0%, and deformed grains reducing to 48.6%. Concurrently, KAM values demonstrate a progressive radial gradient (center-to-edge increase), inversely correlated with the decreasing grain size trend. Notably, radial-direction grains exhibit finer dimensions compared to axial counterparts (Figure 6), likely attributed to radial compressive stresses elongating grains along the axial direction, thereby enhancing apparent refinement when observed radially.

Additionally, the edge region undergoes greater deformation, where high-stress conditions drive a rapid increase in dislocation density, thereby providing thermodynamic driving forces for DRV and DRX. This premise is corroborated by the lower average KAM value observed at the edge compared to the mid-radius zone. The reduced KAM at the edge arises from dislocation annihilation via intensified DRV and DRX. Concurrently, the higher fraction of DRX grains and smaller grain size in the edge region confirm enhanced DRX activity. In summary, deformation stress enhances dislocation density and stored energy within the material, synergistically advancing both DRV and DRX through localized thermal activation.

## 4. Discussion

### 4.1. Strengthening Mechanism

Rotary swaging serves as an effective method for alloy strengthening. During the swaging process, the specimen undergoes repeated short-range radial impact stresses, inducing massive dislocation accumulation as illustrated in Figure 8. The high dislocation density provides driving forces for DRV and DRX, facilitating the formation of subgrain boundaries and new grain boundaries, which significantly reduce grain size and enhance grain refinement strengthening, as shown in Figure 7.

**Figure A1:**
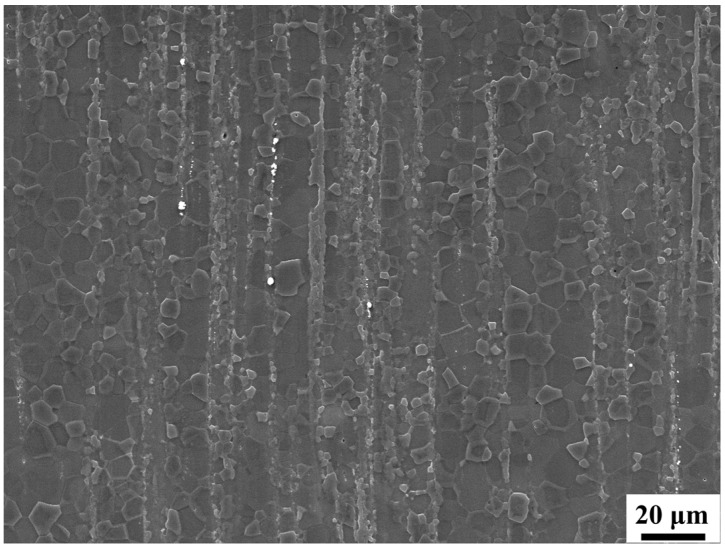
Secondary electron scanning image of the as-extruded Mg-0.7Sm-0.3Zr alloy.

In this study, Sm exhibits limited solubility in magnesium and may potentially form the Mg_41_Sm_5_ secondary phase; however, no such phase has been observed in previous investigations [26]. Zr is virtually insoluble in magnesium, and its particles in the extruded Mg-0.7Sm-0.3Zr alloy can be seen in Appendix A Figure A1. As illustrated, the alloy contains very few secondary phases, and calculations based on established equations indicate that the strengthening contribution from these phases is negligible. Therefore, to simplify the analysis, the effect of secondary phase strengthening is not considered in this study. Concurrently, after one swaging pass, numerous tensile twins develop within the alloy. These twins significantly alter crystallographic orientations, promoting the formation of a strong (0001) [1$\bar{1}$00] fiber texture, thereby contributing to texture hardening. Therefore, the yield strength (σ_y, cal_) of the Mg-0.7Sm-0.3Zr alloy after rotary swaging can be estimated through dislocation strengthening (σ*_ρ_*), grain refinement strengthening (σ_gb_), and texture strengthening (σ_tex_) as follows [36,37]:

(2) $$\sigma_{y,cal}=\sigma_{0}+\sigma_{gb}+\sigma_{\rho }+\sigma_{tex}$$

where σ_0_ is the lattice friction stress (typically ~50 MPa [38,39]). σ_gb_ can be calculated by the following equation [37]:

(3) $$\sigma_{gb}=f_{DRX}k{d_{DRX}}^{-1/2}+f_{non-DRX}k{d_{non-DRX}}^{-1/2}$$

To accurately estimate the grain refinement strengthening effects across different swaging passes, DRX grains and non-dynamically recrystallized (non-DRX) grains were distinguished. Their respective proportions (*f*) and corresponding average grain sizes (d) were calculated, as summarized in Table 2. The Hall–Petch slope (*k*) [14], adopted as 205 MPa·μm^1/2^ in this study.

$\sigma_{\rho }$ can be calculated as follows [40]:

(4) $$\sigma_{\rho }=M\alpha Gb\sqrt{\rho_{GND}}$$

where M is the Taylor factor (typically 2.1 [41]); G denotes the shear modulus of magnesium (~16.6 GPa for Mg alloys [39]); α is a temperature-dependent constant with a value of 0.2 [39]; b represents the Burgers vector magnitude (~0.32 nm for Mg alloys [41]); and *ρ*_GND_ refers to the GND density.

Since basal slip typically dominates deformation in magnesium alloys, the texture strengthening component (σ_tex_) primarily reflects the alloy’s resistance to activation of the basal slip system during deformation [42]. This can be estimated as [42] follows:

(5) $$\sigma_{tex}=\frac{\tau_{basal}}{m_{basal}}$$

Here, τ_basal_ represents the critical resolved shear stress (CRSS) for basal slip, which ranges between 2 and 8 MPa in Mg alloys [43], and m_basal_ denotes the average Schmid factor for basal slip.

Contributions of different strengthening mechanisms to the Mg-0.7Sm-0.3Zr alloy bars under varying swaging passes are summarized in Table 2. As shown, the experimentally and calculated TYS exhibit excellent agreement. Grain refinement strengthening dominates the strengthening mechanisms during the swaging process of the Mg-0.7Sm-0.3Zr alloy. With increasing swaging passes, the average DRX grain size remains relatively stable, while the non-DRX grain size undergoes a more pronounced reduction. Notably, dislocation strengthening contributes 47 MPa after the first swaging pass and reaches a maximum of 55 MPa after 3 passes, indicating rapid dislocation multiplication in the alloy, consistent with its high deformability. Due to the preferential activation of basal slip in Mg alloys during deformation, a basal texture forms. Consequently, with progressive deformation, the basal planes gradually align parallel to the axial direction, resulting in reduced average Schmid factor (SF) for basal slip and progressively enhanced texture strengthening effects.

### 4.2. Grain Refinement Mechanisms

**Figure 12:**
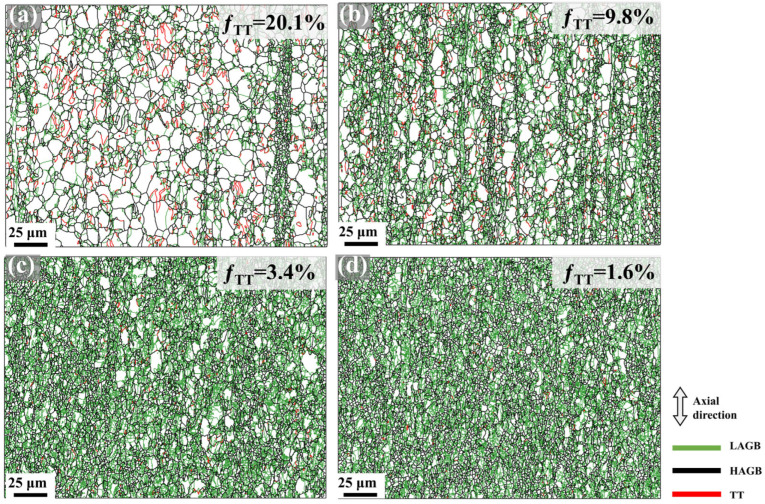
Grain boundary maps in Mg-0.7Sm-0.3Zr alloy bars during multi-pass rotary swaging: (**a**) first pass; (**b**) second pass; (**c**) third pass; (**d**) fifth pass.

Based on the characterization results in Figure 6, tensile twins are predominantly observed within grains only during the initial swaging passes (first and second), while they become scarcely detectable in later passes. Figure 12 displays the grain boundary maps and the area fraction of tensile twins (*f*_TT_) of the Mg-0.7Sm-0.3Zr alloy after varying swaging passes. After the first pass, abundant tensile twins emerge in the alloy, with an *f*_TT_ of 20.1%, primarily driving early-stage grain refinement. Chen et al. [21] observed nanoscale grains at tensile twin intersections, attributing this refinement mechanism to stress concentration at twin boundaries. As swaging progresses, the proportion of LAGBs steadily increases, while the fraction of tensile twins decreases sharply, from 20.1% down to just 1.6%. This reduction stems from three factors: (i) the gradual growth of pre-existing twins to consume parent grains, or (ii) lattice rotation under high deformation, causing twin boundaries to lose their characteristic misorientation; (iii) the formation of a strongly basal fiber texture and small grain size suppress tensile twin activation under radial compressive stresses [44], preventing further twin nucleation. Although tensile twins are absent in later swaging passes, they exhibit pronounced activity during initial deformation stages. Tensile twinning preferentially activates in larger grains, with the formation of twin boundaries contributing to overall grain refinement. Moreover, propagation of tensile twins through parent grains further subdivides original coarse grains, amplifying the refinement effect. Thus, tensile twinning serves as the dominant grain refinement mechanism in early-stage rotary swaging.

**Figure 13:**
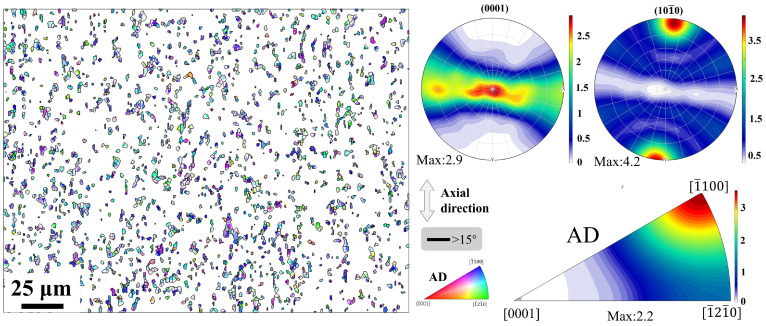
IPF maps, (0001) PF, (10$\bar{1}$0) PF and IPF of recrystallized grains following the fifth rotary swaging pass.

In later swaging passes, results from Figure 7, Figure 8 and Figure 12 indicate that intense strain accumulation triggers a sharp rise in dislocation density, promoting DRX to refine grain sizes. Figure 13 presents IPF maps, PF and IPF characterizing the newly formed DRX grains after the fifth pass of rotary swaging. Notably, newly formed DRX grains retain fiber texture orientations, confirming that continuous DRX (CDRX) dominates the recrystallization process. Statistical analysis in Table 2 demonstrates nearly invariant average sizes for DRX grains but progressive refinement in non-DRX counterparts. These observations collectively suggest that CDRX primarily proceeds via strain-induced grain boundary bulging nucleation [45]. At triple junction boundaries with elevated dislocation density, stress-induced migration of original grain boundaries generates new grains. Consequently, parent grain dimensions diminish progressively with increasing swaging passes, while the volume fraction of new DRX grains grows without significant size variation, as evidenced by the data trends in Figure 7 and Table 2.

**Figure 14:**
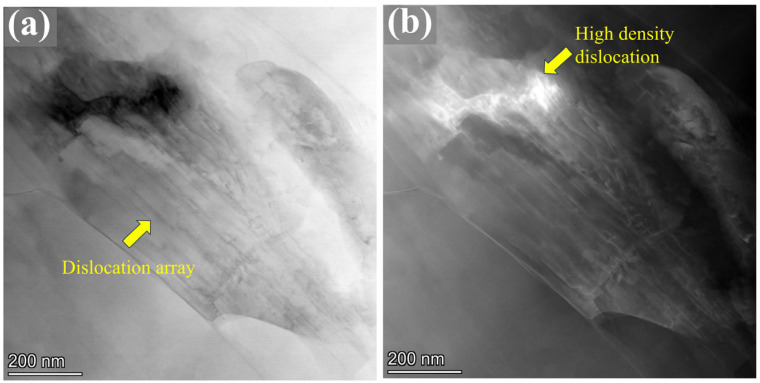
TEM micrographs of the Mg-0.7Sm-0.3Zr alloy after five passes of rotary swaging deformation: (**a**) bright-field image, (**b**) dark-field image.

Additionally, TEM characterization was performed on the Mg-0.7Sm-0.3Zr alloy after five passes of rotary swaging, as shown in Figure 14. The severe plastic deformation resulted in the formation of submicron-sized grains with a size of approximately 600 nm. A large number of dislocation arrays were observed within the grains, as indicated by the yellow arrows in Figure 14a. Moreover, regions with a high density of dislocation pile-ups were also identified, as shown in Figure 14b. Studies [46] have shown that in magnesium alloys, when the stored strain energy becomes sufficiently high, these dislocation arrays can promote the occurrence of DRX. The substantial multiplication of dislocations induced by the intense deformation during rotary swaging, coupled with the increase in internal temperature of the bar, leads to the formation of fine and clean DRX grains.

According to the radial microstructure characterization results in Figure 10 and Figure 11, the edge region of the swaged bar exhibits smaller average grain sizes, higher dislocation densities, and a greater fraction of recrystallized grains compared to the central region, leading to enhanced grain boundary and dislocation strengthening effects at the edge. The hardness of the edge region after two swaging passes is significantly higher than that of the center, as shown in Figure 4. With increasing swaging passes, this edge-to-center hardness disparity gradually diminishes, likely due to restrictions on intra-grain dislocation accumulation [47]. After achieving higher grain boundary and dislocation strengthening at the edge, deformation stress progressively shifts to the center. Consequently, grain refinement during swaging of the Mg-0.7Sm-0.3Zr bar initiates at the edge and extends inward toward the central region.

## 5. Conclusions

This study systematically investigates the microstructure and mechanical properties of Mg-0.7Sm-0.3Zr alloy bars subjected to multi-pass rotary swaging at room temperature, with a focus on the strengthening mechanisms and grain refinement processes induced by swaging. The primary conclusions are as follows:

1. Rotary swaging at room temperature significantly enhances the hardness and strength of the alloy. After 5 passes, the radial average hardness reaches 71.6 HV, with TYS and UTS increasing to 292 MPa and 302 MPa, respectively, while elongation decreases to 11.6%.
2. The microstructure of the Mg-0.7Sm-0.3Zr alloy is markedly refined following swaging. The average grain size decreases from 4.37 μm to 2.02 μm. The proportion of LAGBs and dislocation density progressively increase with swaging passes. After 2 swaging passes, the alloy exhibits a pronounced radial gradient structure from the surface to the core regions, characterized by progressively decreasing grain sizes and increasing KAM values toward the center.
3. The improvement in strength results from a combination of grain refinement strengthening, dislocation strengthening, and texture strengthening. Grain refinement plays a dominant role, while dislocation strengthening reaches saturation after two passes, showing negligible variation in subsequent passes.
4. The grain refinement in Mg-0.7Sm-0.3Zr alloy during swaging occurs through two distinct mechanisms. In early passes, the extensive activation of tensile twinning partitions and segments the initial coarse grains, facilitating refinement. In the later stages, stress-induced grain boundary bowing generates fresh DRX grains that further subdivide the deformed coarse grains, thereby enhancing the overall refinement.

## Figures and Tables

**Table 1:** The bar diameter and true strain under different passes of rotary swaging.

Samples	Bar Diameters (mm)	True Strain (%)
Extrusion	19.2	0
1st pass	18.9	1.6
2nd pass	18.4	4.3
3rd pass	17.2	11.0
5th pass	14.8	26.0

**Table 2:** Contribution of each strengthening mechanism in Mg-0.7Sm-0.3Zr alloy bars with different rotary swaging passes.

Samples	d_DRX_ (μm)	d_non-DRX_ (μm)	m_basal_	σ_gb_ (MPa)	σ*_ρ_* (MPa)	σ_tex_ (MPa)	σ_y, cal_ (MPa)	σ_y_ (MPa)
1P	1.23	3.86	0.28	115	47	17	229	225
2P	1.17	3.12	0.22	127	53	23	253	252
3P	0.99	2.54	0.20	142	55	25	272	280
5P	0.91	2.28	0.16	155	54	32	291	292

## Data Availability

The original contributions presented in this study are included in the article. Further inquiries can be directed to the corresponding author.

## Abbreviations

The following abbreviations are used in this manuscript:

HV	Vickers Hardness
MPa	Megapascal
RE	Rare Earth
SPD	Severe Plastic Deformation
UTS	Ultimate Tensile Strength
TYS	Tensile Yield Strength
EL	Elongation
DRX	Dynamic Recrystallization
DRV	Dynamic Recovery
EBSD	Electron Backscatter Diffraction
ED/AD	Extrusion Direction/Axial Direction
LAGB	Low-Angle Grain Boundaries
HAGB	High-Angle Grain Boundaries
GND	Geometrically Necessary Dislocation
GOS	Grain Orientation Spread
IPF	Inverse Pole Figure
KAM	Kernel Average Misorientation
CRSS	Critical Resolved Shear Stress
SF	Schmid Factor
CDRX	Continuous Dynamic Recrystallization
